# Improved data quality and statistical power of trial-level event-related potentials with Bayesian random-shift Gaussian processes

**DOI:** 10.1038/s41598-024-59579-2

**Published:** 2024-04-17

**Authors:** Dustin Pluta, Beniamino Hadj-Amar, Meng Li, Yongxiang Zhao, Francesco Versace, Marina Vannucci

**Affiliations:** 1 Department of Biostatistics and Data Science, Augusta University, Augusta, GA 30912 USA; 2 Department of Statistics, Rice University, Houston, TX 77005 USA; 3 Department of Statistics and Computer Science, University of Michigan, Ann Arbor, MI 48109 USA; 4 Department of Behavioral Science, MD Anderson Cancer Center, Houston, TX 77030 USA

**Keywords:** Statistics, Cognitive neuroscience

## Abstract

Studies of cognitive processes via electroencephalogram (EEG) recordings often analyze group-level event-related potentials (ERPs) averaged over multiple subjects and trials. This averaging procedure can obscure scientifically relevant variability across subjects and trials, but has been necessary due to the difficulties posed by inference of trial-level ERPs. We introduce the Bayesian Random Phase-Amplitude Gaussian Process (RPAGP) model, for inference of trial-level amplitude, latency, and ERP waveforms. We apply RPAGP to data from a study of ERP responses to emotionally arousing images. The model estimates of trial-specific signals are shown to greatly improve statistical power in detecting significant differences in experimental conditions compared to existing methods. Our results suggest that replacing the observed data with the de-noised RPAGP predictions can potentially improve the sensitivity and accuracy of many of the existing ERP analysis pipelines.

## Introduction

**Figure 1:**
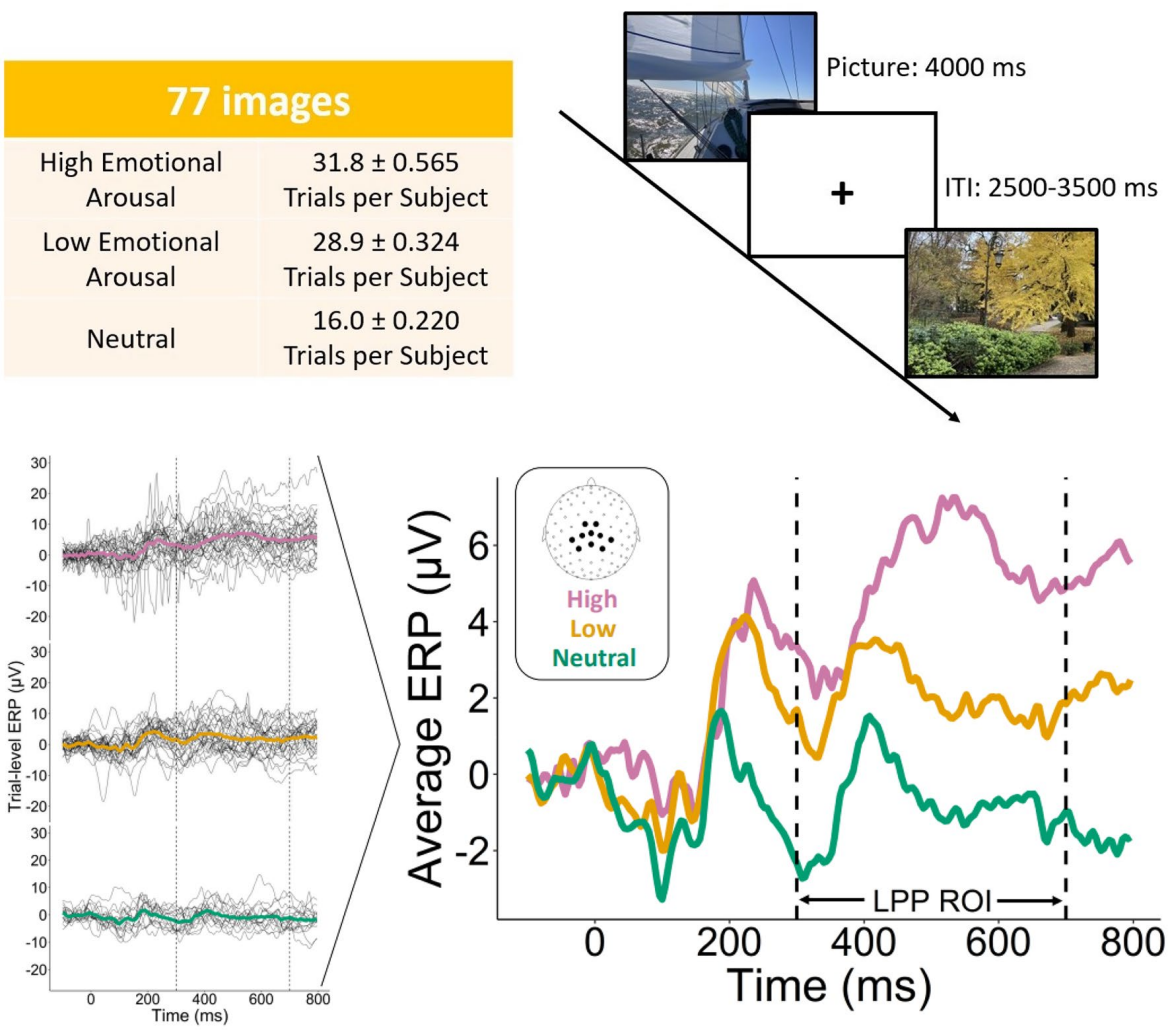
Trial-level ERP data modeling. In each experimental trial, a subject is shown an image drawn from a database consisting of images which are classified as high emotional arousal, low emotional arousal, and emotionally neutral. Trial-level data from ERP experiments is often summarized into condition-specific average curves for further analysis. The late positive potential (LPP) window is defined as ranging from 300 to 700 ms after stimulus presentation.

In studying the association of electroencephalogram (EEG) recordings and cognitive and behavioral outcomes in humans, researchers often focus on the study of event-related potentials (ERP), i.e., specific EEG segments time-locked to cognitive events or experimental stimuli^1^. Due to the substantial noise present in EEG recordings and the relatively weak signal, ERP analyses often focus on group-level effects that can be estimated from average ERPs^2^. For example, in the case study we consider in this paper, the goal is to detect differences in response to images with differing levels of emotional arousal, e.g. highly arousing, mildly arousing, or unarousing (Fig. 1). A commonly used analysis technique for this data summarises a specified time-window in the form of average amplitudes (i.e., voltage) to be analysed, for example, via one-way repeated measures ANOVAs across groups of subjects and/or conditions^3^, or with an empirical bootstrap procedure^4,5^. Consideration of the average ERP in statistical analyses is often necessary due to a lack of available tools to model trial-level ERP features. Consequently, the potential impact of trial-level features on the estimation of the average ERP is often ignored or mitigated through pre-processing procedures^6,7^. Moreover, while there has been some study of within-subject variability of ERP characteristics, such as latency and amplitude, most results regarding the association of these features with cognitive and behavioral outcomes have been established only at the group level^8–11^.

Early approaches for the analysis of trial-specific latencies of ERPs were presented in^12^ and^13^, and later extended to include estimation of trial-specific amplitudes by^14^. This initial work on trial-specific latency and amplitude estimation lead to the development of a class of so-called Variable Signal plus Ongoing Activity (VSPOA) models, a general framework for the analysis of brain potentials that assumes that the observed signal for each trial is a linear combination of a fixed number of components, each of which may be shifted by some trial-specific latency, plus trial-specific ongoing background activity. The differentially variable component analysis (dVCA) introduced by^15^ is a Bayesian VSPOA that provides maximum a posteriori estimates of trial-specific latencies, amplitudes and component waveforms. In an effort to improve upon dVCA^16^, proposed the analysis of single-trial ERP and ongoing activity (ASEO) method, which assumes autoregressive ongoing brain activity, rather than white noise, as in dVCA, and which allows for estimation of latency on a continuous scale, whereas dVCA restricts latencies to be integer multiples of the sampling interval. However, both dVCA and ASEO suffer from the need to initialize the number of components and component waveforms. In practice, it is common to inspect the average ERP of the data and initialize the components as segments of the average ERP^17,18^. However this data-driven initialization may impact the resulting inference and bias the estimated components toward the average ERP. In addition to VSPOA models, a number of other methods for trial-level ERP analysis have been proposed, including spatial and wavelet filtering^19,20^, graph-based variability^21,22^, and linear mixed models^23,24^. While these methods are effective in obtaining estimates of trial-specific parameters and component waveforms, they are often not suitable for inference of arbitrary single-trial characteristics, and may suffer from restrictive assumptions regarding waveform structure and initialization, the single-trial noise model, and incorporation of prior scientific beliefs.

Methods described above have been mainly developed for the analysis of trial-level ERP signal, that is, single trial EEG data collected and processed according to time-locked stimuli. In recent years, there has been renewed interest in trial-by-trial analyses of raw EEG signals, i.e., not time-locked, using, e.g., support vector machines^25,26^, spatial network discriminant analysis^27^, topological data analysis^28^, and deep neural networks^29–31^. In contrast to model-based trial-level ERP analysis, these methods are primarily for the prediction of trial conditions from single-trial EEG waveforms, rather than inference of ERP components and trial-specific quantities of interest. A comparison of the performance of logistic regression, support vector machines, and neural networks in EEG signal classification and ERP detection in different signal-to-noise ratio (SNR) settings is given in^32^. While these predictive algorithms have found success in application, particularly for training of brain-computer interface systems^33–35^, it is often difficult to identify and interpret the discriminating features.

Motivated by the need for rigorous, flexible, and interpretable methods for trial-level analysis of ERP data, we developed the Random Phase-Amplitude Gaussian Process (RPAGP) modeling framework, which assumes that individual trials are generated as a common structural signal transformed by a trial-specific amplitude and phase shift plus ongoing brain activity. In the proposed RPAGP framework, a form of VSPOA model, the unknown signal is modeled via a Gaussian Process (GP) prior and an autoregressive process is assumed for the ongoing brain activity. We set priors on the trial-specific model parameters and design an efficient algorithm for posterior inference. In contrast to methods for group-level analysis of ERP data, our method produces estimates and uncertainty quantification of the structural signal and trial-specific characteristics, along with posterior distributions for trial-specific signals. This feature makes it possible for RPAGP to improve the sensitivity and accuracy of existing ERP analysis pipelines by replacing the observed data with the de-noised model predictions. Here we use the data from^36^ to illustrate the use of RPAGP for testing of trial-level characteristics. Previous group-level analysis of this data has shown significance evidence that the amplitude of the late positive potential (LPP, see Fig. 1) is proportional to the level of arousal of the image, a result which is corroborated by the existing literature^3,37–41^. We reproduce such result with a trial-level analysis of single subject data. We also show how data quality and statistical power can potentially be greatly improved via our model-based de-noising.

Our proposed model falls within the general class of VSPOA models, as discussed above. Even though motivated by neuroscientific beliefs about the structure and characteristics of ERP signals, it has been difficult to develop a practical statistical implementation of these general models, due to the challenge of specifying the form of the component curves a priori in a manner that admits valid statistical inference. The closest construction to the proposed RPAGP model is the ASEO method of^16^ which assumes a VSPOA model with autoregressive ongoing activity and uses an iterative numerical algorithm for inference that depends on extensive use of the Fourier transform. To the authors’ knowledge, the most readily available implementation of ASEO is in the FieldTrip MATLAB toolbox as part of the “event timing analysis” routine^42^. However, running ASEO via FieldTrip requires a priori specification of the number of components, and either the initial latencies of each component or initial estimates of the component waveforms through a decomposition of the average ERP, which in turn requires choosing the time window associated with each component. More importantly, while providing accurate point estimates of model parameters and reconstruction of component waveforms, the ASEO algorithm does not provide uncertainty quantification of these estimates, and so it is limited for statistical inference. On the contrary, RPAGP allows a flexible construction via the use of Gaussian process priors, which provides full inference on trial-level latency, amplitude, and the structural signals associated with each experimental condition. We demonstrate the proposed model is practical for application to data produced by most current ERP experiments, and does not require subjective initialization of component curves. In the Supplementary Material A1 we use simulated data to further investigate performance of the proposed RPAGP model to reconstruct the structural signal.

## Results

We consider the data analysed in^36^ to study emotional processes. In each experimental trial, a subject is shown an image drawn from a database of images^43–45^. For the analyses of this paper we included 32 images rated as highly arousing, 29 rated as low arousing, and 16 rated as emotionally neutral (Fig. 1, see “Methods”). Scientific interest is primarily regarding the mean of the task-related signal over the LPP window, which is defined as ranging from 300 to 700 ms after stimulus presentation. Previous analyses have shown that the magnitude of the LPP mean averaged over trials and subjects is positively associated with the level of arousal of the stimuli^3^. We re-analysed the data by fitting the proposed RPAGP model (see “Methods”) to the trial-level data in the LPP window, standardized to [0, 1] with increments $\Delta t = 1/224$, and assuming a common structural signal across all trials.

### Single subject analysis

**Figure 2:**
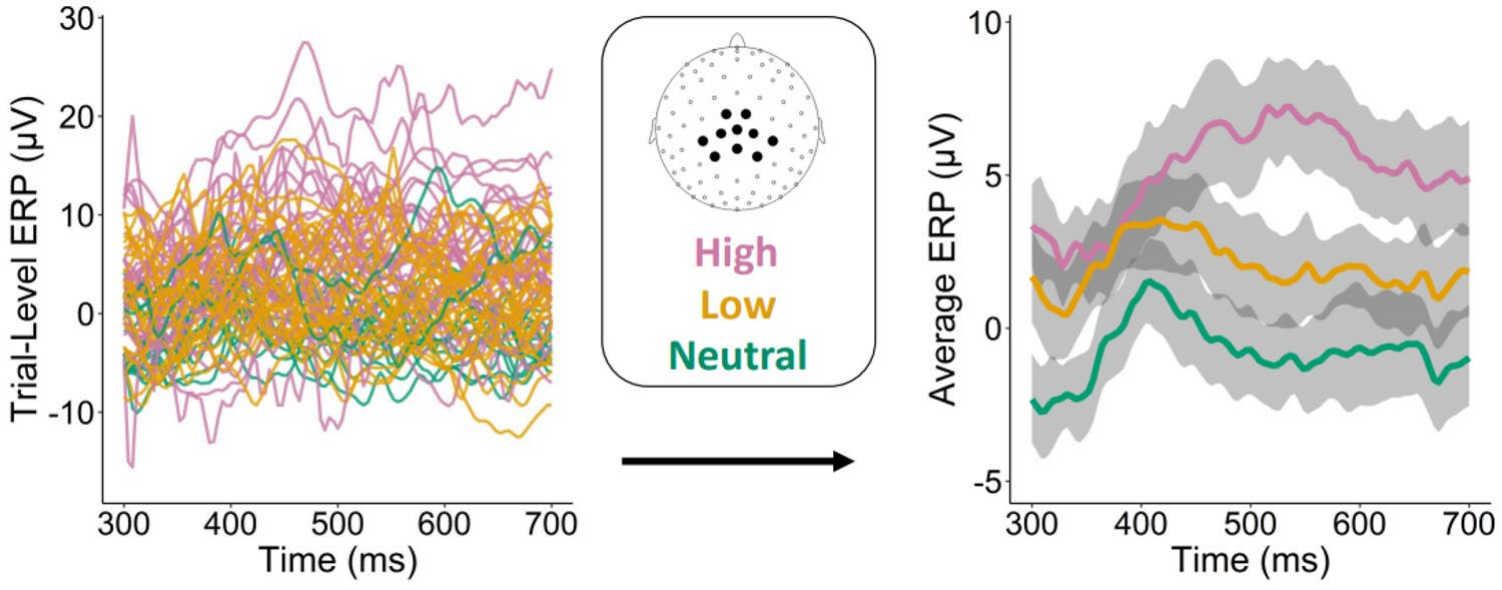
Single-subject analysis of trial-level ERP signals. (left) Raw trial data from a single subject. (right) Means by condition show the expected relationship, but the associated 90% confidence bands, obtained by the empirical bootstrap, are wide and do not clearly suggest a statistically significant difference between the low and neutral categories.

**Figure 3:**
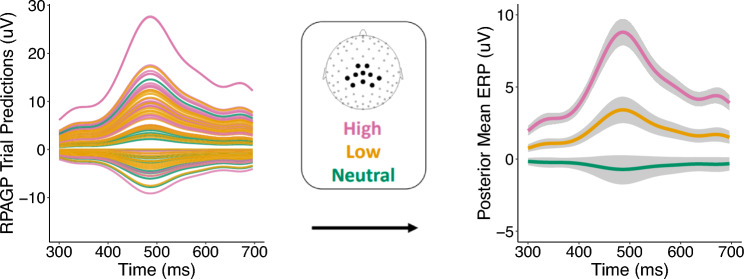
Inference from RPAGP on single-subject trial-level ERP signals. (left) The temporally-aligned RPAGP trial predictions, estimated via the posterior median, extract the task-related signal from the trial-specific noise. (right) Means by condition obtained by averaging the trial estimates. The 90% credible bands provide strong evidence of a difference in means among conditions.

In order to illustrate the application of the RPAGP model we first present results from the analysis of one subject. Figure 2 shows the data (left panel), together with empirical means, averaged by condition, and 90% bootstrap confidence intervals (right panel) computed by a stratified bootstrap procedure (see “Methods”). The 90% confidence bands are used here for better visualization of the condition estimates, as the 95% bands would obscure the differences. The empirical estimates suggest differences in the LPP means by condition, but it is not clear if these differences are statistically significant. As is evident from the data, there is substantial noise present in single-trial ERP data, due to on-going brain activity and idiosyncratic fluctuations. These noise components are not accounted for in the empirical bootstrap method, resulting in wide confidence bands. On the contrary, Fig. 3 shows the temporally-aligned RPAGP trial predictions $\tilde{y}_i(t)$, estimated via the posterior median, obtained by fitting the proposed PGP model (left panel) and the RPAGP model means obtained by averaging the aligned trial estimates by condition, together with the 90% credible intervals (right panel). We note that the posterior estimates of the means by condition calculated from the RPAGP model exhibit similar shape and scale to the empirical estimates, but with much less uncertainty relative to the empirical bootstrap method; the separation of estimated response by condition is clear from the model fits, but less apparent when considering the bootstrap estimates. Application of RPAGP allows removal of the noise and latency components, which results in narrower credible bands and a more powerful detection of differences among conditions.

One may notice that the empirical means and posterior mean ERPs in Figs. 2 and 3 look markedly different, particularly in the neutral condition. This is the result of the RPAGP modeling assumptions and differences in the methods of estimation. In the empirical approach, each condition is considered individually, and the respective trials are averaged to obtain a condition-specific empirical ERP estimate. With this approach, structural characteristics of the background activity may be present in the condition averages. Furthermore, subsetting the trials by condition can result in larger variance estimates. In comparison, the model-based RPAGP estimates are obtained under the assumption that the structural shape of the ERP is common across all conditions. Therefore, the higher voltage from 300 to 500 ms observed in the Neutral condition, for example, is likely not captured by the model as it is not consistently present in the other two conditions. Furthermore, since amplitudes of the Neutral condition trials are close to zero, the posterior mean curve will be close to the zero curve, and could obscure features in the Neutral condition even if it were significantly present in all conditions. The zeroing of the Neutral condition curve is not unexpected, as the assumption of the Neutral condition images is that they produce a small or no LPP response. This also explains why we see little difference in the High condition curves, as the signal is strongest in this condition and structures from background activity are less prominent in the empirical mean relative to the Neutral condition. Plots of raw trial data and means by condition, together with the temporally-aligned RPAGP trial posterior estimates and means by condition, for 4 additional subjects, are shown in the Supplementary Material A1.

**Figure 4:**
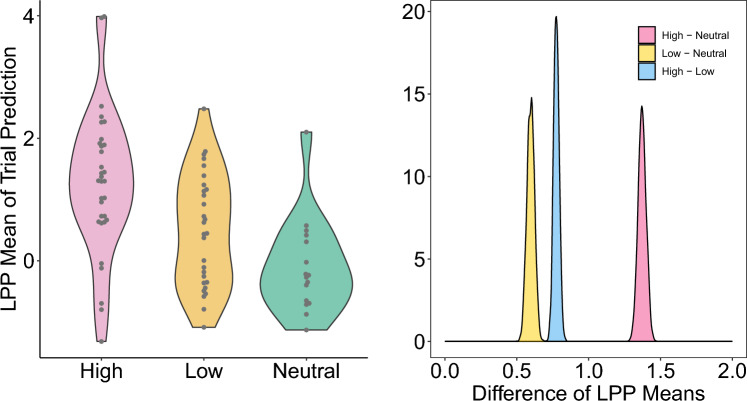
Inference on LPP means. (left) The posterior densities of the RPAGP estimates of trial LPP means by condition suggest that the selected subject exhibits the expected relations among the LPP means on average. There is also substantial variability apparent in the LPP means between trials within the same condition. (right) The posterior densities of the differences in LPP means are tightly concentrated and significantly greater than zero, in accordance with the expected relationships among conditions.

Posterior densities of the trial LPP means (Fig. 4, see “Methods”) show that trials from the selected subject follow the expected trend on average, with a large amount of variability in the LPP means of individual trials, resulting in distributions by condition with substantial overlap. Despite the wide range of trial LPP means, the posterior distributions of the difference of LPP means among conditions are positive and tightly concentrated away from zero, clearly supporting the LPP hypotheses $\mu _{LPP}^{High}> \mu _{LPP}^{Low} > \mu _{LPP}^{Neutral}$. The largest difference in mean is exhibited by the comparison of the High and Neutral conditions, with an estimated difference in means (90% CI) of 1.37 (1.33, 1.42); the High–Low and Low–Neutral differences were similar, with means and CIs of 0.78 (0.75, 0.81) and 0.60 (0.55, 0.64) respectively. For comparison, the empirical bootstrap procedure also finds the largest difference between the High and Neutral categories with an estimated difference in means (90% confidence interval) of 1.41 (0.97, 1.84). The Low–Neutral and High–Low differences are estimated as 0.61 (0.22, 0.98) and 0.70 (0.24, 1.15) respectively. While each of the three comparisons are found to be significantly greater than zero and with similar point estimates for both methods, we observe that the bootstrap confidence intervals are wider than the model credible intervals.

**Figure 5:**
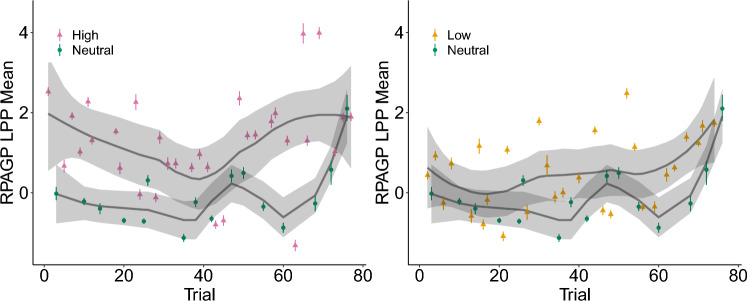
Trend analysis of the LPP mean estimates. Longitudinal plots of trial-specific LPP means (points), posterior 90% credible intervals (error bars), loess smoothed curves (solid lines), and loess standard error bands (shaded regions). These plots illustrate trial-level variability and can reveal trends over the course of the experiment.

Finally, Fig. 5 shows the RPAGP estimates of trial LPP means ordered sequentially over the course of the experiment. These plots can be useful to understand the variability of the characteristic of interest over the course of the experiment. We note that both the Low and High trials tend to have larger mean than the Neutral trials and that there is greater variability present in both emotionally arousing categories compared to neutral images, with the High condition images showing the greatest variability overall.

### Impact on data quality

Next, we present results from the application of the RPAGP model to ERP data recorded from 161 subjects participating in the LPP emotional response image experiment of^36^. Table 1 shows the proportion of subjects satisfying the expected LPP mean relationships among conditions for the RPAGP method, the empirical bootstrap (EMP) method and a standard ANOVA test performed on the empirical means from the original trial data, averaged by condition. We also report results we obtained by applying the ASEO algorithm via the Fieldtrip MATLAB toolbox^42^ to obtain estimates of the trial-specific signals, from which the ASEO trial-specific LPP means were computed. Confidence intervals of the differences in LPP means by condition were constructed by following the empirical bootstrap procedure but replacing the observed data with the ASEO trial-specific estimates. Significance of difference in LPP means was determined from these confidence intervals as with the empirical bootstrap procedure. On all three tests, RPAGP finds a much higher proportion of subjects satisfying the expected mean relationships compared to ASEO, EMP, and ANOVA, with RPAGP detecting 86.3%, 75.8%, and 70.2% of subjects showing significant differences for the High–Neutral, High–Low, and Low - Neutral tests, respectively. This is compared to 45.3%, 26.7%, 18.6% for ASEO; 44.1%, 29.2%, and 22.4% for EMP; 42.9%, 28.8%, and 16.8% for ANOVA, on the same tests, respectively. We note that RPAGP shows joint significance on all three comparisons for 52.2% of subjects, compared to only 6.8% of subjects detected by EMP. For a further examination of test performance, we focus on a comparison of RPAGP and EMP, since ASEO and ANOVA show lower proportions of significant subjects on all three tests.

We also tested whether our method increases the risk of detecting false positives. For each subject, we randomly divided the trials of each condition into two groups and performed an ANOVA test for the difference in means. We repeated this random split 1000 times for each condition and calculated the percentages of significant tests. With the modeled data, the average of significant results was 4.3%, 4.5% and 4.6% for the High, Low and Neutral conditions, respectively. For comparison, applying the same procedure to the raw ERP data resulted in 4.5%, 4.5% and 4.4% significant differences for the High, Low and Neutral conditions, respectively. We conclude that our method substantially increases the chance of detecting a true positive while not increasing the probability of false positives.

**Table 1:** Impact on data quality.

	High–neu.	High–low	Low–neu.	All 3 tests
RPAGP	0.863	0.758	0.702	0.497
ASEO	0.453	0.267	0.186	0.031
EMP	0.441	0.292	0.224	0.068
ANOVA	0.429	0.288	0.168	0.038
All 4 methods	0.429	0.267	0.168	0.031

**Figure 6:**
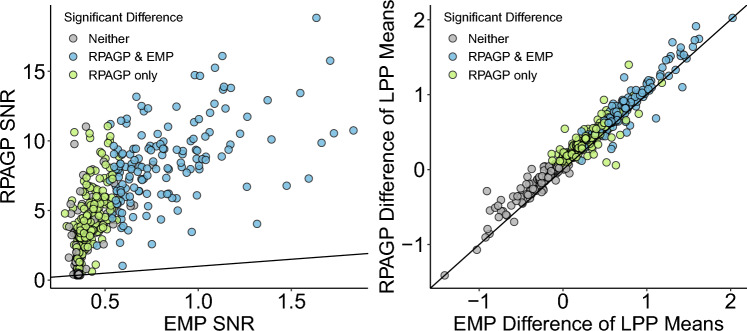
Improvement on data quality of the RPAGP denoising. (left) Comparison of the signal-to-noise ratio (SNR) of the model predictions (RPAGP) against the empirical means (EMP). The de-noising process of the model improves the SNR for all but one trial, with a 15-fold improvement on average. (right) The RPAGP and empirical estimates of the differences in LPP means by condition essentially agree for all trials, thus the improvements in statistical power for RPAGP primarily result from the removal of noise.

**Figure 7:**
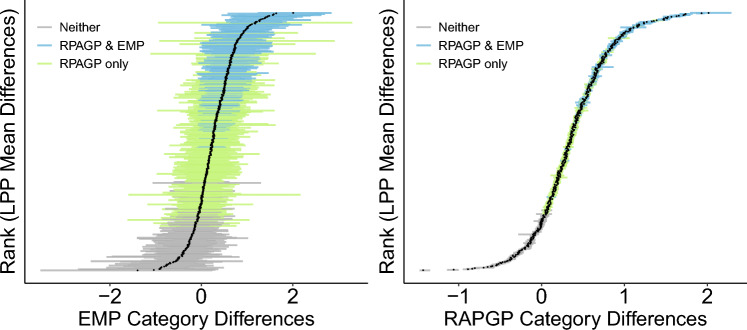
Differences in LPP means between categories. The 90% credible intervals for differences in LPP means between categories produced by RPAGP (right) are substantially narrower than the 90% confidence intervals obtained via empirical bootstrap (left).

Proportion of subjects giving expected results. RPAGP detects a substantially higher proportion of subjects following the expected mean relationship among categories compared to ASEO, EMP and ANOVA. This is primarily due to the de-noising process of the model, which reduces uncertainty about the estimates for the differences among conditions, compared to the other methods that do not account for the structured and idiosyncratic noise present in ERP data (see Figs. 6 and 7 and text).

To quantify the practical effect of the model de-noising provided by RPAGP in the LPP experiment, Fig. 6 illustrates the change in signal-to-noise ratio (SNR, see “Methods”) and a comparison of the point estimates of difference in the means by condition, for the RPAGP and EMP methods, and Fig. 7 shows a comparison of the CI widths for these estimates. The RPAGP model greatly improves the SNR compared to the EMP method, with a 15-fold increase in SNR on average. This improvement in SNR could result from increases in the estimated differences, decreases in the widths of the corresponding CIs, or a combination thereof. Examination of the point estimates for differences in means produced by RPAGP and EMP shows that they are essentially equivalent (Fig. 6), but, as seen in Fig. 7, the CIs produced by EMP are much wider than those produced by RPAGP. Thus the improvement in SNR is entirely due to the narrower confidence intervals associated with the RPAGP estimates, rather than increased point estimates. The additional cases detected by RPAGP but not EMP (bright green) are primarily those with small point estimates, but also include a number of cases with relatively large point estimates. RPAGP detects a significant relationship for all but one test, with a point estimate of differences among the means by condition above 0.135, and shows consistent CI widths across all subjects and tests. In contrast, EMP fails to identify a significant relationship even for some comparisons with estimated differences; the EMP CIs are substantially larger than the RPAGP CIs across all tests and subjects, and much greater variability of CI width is evidence in the EMP CIs. This stark difference in CIs is due to the separation of signal and noise in the RPAGP model, making the resulting error estimates less susceptible to subject- and trial-specific variations that are not related to the stimuli.

For further evidence, we calculated the Standardized Measurement Error (SME, see “Methods”), a measure of (subject-level) data quality proposed by^46^ for averaged ERPs, using both the raw data and the RPAGP trial predictions. Results showed that the RPAGP de-noising decreases the SME compared to the raw data, but also that the resulting change in SME is relatively small, with the RPAGP trial predictions showing an average decrease (standard error) of $0.039 \pm 0.062$ relative to an average SME of 0.555 computed over the raw data and model predictions. This close correspondence in SME from the raw data and trial predictions indicates that the model does not substantially reduce inter-trial variability relative to the raw data. In conjunction with the results on improvement in SNR from application of RPAGP, the correspondence of SME suggests that the improvement of RPAGP over EMP (Table 1) comes primarily from a reduction in noise of the estimated parameters, rather than from a boosting of the LPP signal (Fig. 6), nor from a reduction in the inter-trial variability.

In the Supplementary Material A1, we report additional investigations into the performances of RPAGP that were performed based on simulated data. Simulated settings, in particular, allow us to calculate Mean Squared Errors (MSEs) between the estimated and the true structural signal. We simulated data for two categories, following the general structure suggested by the real ERP data of our application. Details of data generation are provided in the Supplementary Material A1. We investigated performances for different values of the model parameters $\sigma _{\beta }, \sigma _{\tau }$ and $\sigma _{\varepsilon }$ (see “Methods”) and by averaging results over 50 replicated datasets. Figure A.1 shows the true structural signal *f*, the simulated trial-level data and the RPAGP estimate $\tilde{f}=\widehat{\bar{\beta }f}$, computed as the posterior median of the distribution of the temporally-aligned trial estimates, for one dataset simulated with $\sigma _{\beta }=0.1, \sigma _{\tau }=0.01$ and $\sigma _{\varepsilon }=0.1$ and $n = 30$. By adjusting for the trial-specific latencies, RPAGP is able to provide a more accurate estimate of the true structural signals relative to the empirical means. MSEs obtained by estimating the true structural signal by RPAGP and by the empirical mean of the raw data, and averaged over the 50 replicates, are reported in Table A.1. RPAGP shows substantially smaller MSE compared to EMP in the presence of latency, and is at least as accurate as EMP when no latency is present.

### Improvement in statistical power

Results above have indicated that the denoised signals produced by RPAGP are potentially less noisy and of better quality than the observed trial-level data. Our next question is to whether the availability of the denoised estimates can also result in a reduction of the number of trials needed to power a particular study, with respect to the standard practice of using average curves obtained from raw data. We therefore assessed the power of the RPAGP method for testing the three hypotheses implied by $\mu ^{High}_{LPP}>\mu ^{Low}_{LPP} > \mu ^{Neutral}_{LPP}$ based on the LPP emotional responses data from^36^ and varying number of trials (see “Methods”), for the subject considered in “Single subject analysis”. Results of this analysis for total number of trials $n = 15; 25$, are reported in Table 2 and show that RPAGP has power at least as great as the empirical bootstrap test for all tests and sample sizes considered. Furthermore, RPAGP substantially outperforms the empirical method in testing for differences between the Low and Neutral conditions, which is the test with lowest signal-to-noise ratio.

**Table 2:** Statistical power analysis.

	$n = 15$	$n = 25$
Empirical H-L	0.65	0.9
RPAGP H-L	0.85	1.0
Empirical H-N	0.95	1
RPAGP H-N	1	1
Empirical L-N	0.50	0.50
RPAGP L-N	0.75	0.90

Estimated power in testing for differences in mean amplitudes across conditions for empirical bootstrap and RPAGP on ERP data. RPAGP consistently shows power at least as great as the empirical testing power, with the greatest increase in testing the difference of Low and Neutral conditions.

For further investigation, we performed a two-group power analysis on simulated data with varying numbers of trials and by generating data with different signal-to-noise ratios. Details of the signals generation are given in the Supplementary Materials A1. For each setting, results were averaged over 50 replicated datasets. The estimated powers are given in Table A.2. The results show that the power of the RPAGP test is substantially greater than that of the empirical method in the majority of simulation settings considered, and is universally better than the empirical method for the largest sample size considered ($n = 30$). In the easier settings, with low SNR, RPAGP has estimated power near or equal to 1, compared to 0.35 for the empirical method.

## Discussion

We have developed the Random Phase-Amplitude Gaussian Process (RPAGP) modeling framework, as a Bayesian implementation of a VSPOA model for trial-level analysis of ERP data. The approach assumes ERP trial data are generated by trial-specific phase shifts and amplitude scalings of a common structural signal that is flexibly modeled with a Gaussian process prior. In contrast to methods for group-level analysis of ERP data, our method produces estimates and uncertainty quantification of the trial-specific parameters and the structural signal, in addition to posterior distributions of trial-specific signals. Trial-level models for ERP data offer markedly advantages over average-based approaches. Firstly, adjusting for variations in trial-level characteristics may result in improved ERP signal estimates, as we have demonstrated in the analyses of this paper. This, in turn, leads to increased statistical power for condition-level inferences compared to empirical methods. Furthermore, analysis of the distribution of trial-level ERP features may reveal scientifically relevant patterns across trials within a subject, or across subjects.

The availability of trial-level signal estimates makes it possible for RPAGP to supplement any existing ERP analysis method by replacing the observed data with de-noised model predictions. De-noised trial predictions may be used, without requiring additional processing, to improve the sensitivity and accuracy of existing ERP analysis pipelines, such as averaging the extracted signals over a scientifically relevant time window, perform spectral analysis and/or assist in detecting outlying or erroneous trials that may not have been removed during pre-processing. We have applied RPAGP to data from a study where the interest is in detecting differences in ERP responses to images with differing levels of emotional arousal. Results, in particular, have shown how data quality and statistical power can potentially be greatly improved via our model-based de-noising.

Possible extensions of the RPAGP model presented here could incorporate alternative assumptions regarding the structure of the data, and further expand its range of applications. For example, for clarity of presentation and application to LPP experimental data, we have assumed a common structural signal for all trials, with potential differences among conditions determined solely by latencies or amplitudes. A direct and simple extension of the proposed model is to allow for different structural signals across experimental categories, each modeled as an independent GP prior, for comparison and testing of trial-level features across categories when there is little information regarding the structure of the condition-specific signals. This form of the model can be implemented with minor changes to the Algorithm 1. Furthermore, the most general class of VSPOA models assume that observed data result from a linear combination of multiple component waveforms, each with its own trial-specific phase and amplitude. While identifying the approximate time intervals and shapes of these components for algorithm initialization can facilitate estimation of trial-specific features, as in^16^, building a flexible inferential procedure incorporating these structural assumptions has not yet been developed, to our knowledge. With the proposed RPAGP model as a starting point, a multiple-component trial-level model similar to the framework in^16^ may be possible through further refining of the model priors. Finally, we did consider some analysis and interpretation of trends in the amplitude and latencies over the course of the experiment, given some of the apparent condition differences that can be seen in Fig. 5. However, given the characteristics of the experimental design, these patterns in a single subject are likely spurious. A formal analysis of trends in ERP amplitudes over the course of the experiment using the proposed model is an interesting possible direction for future work.

## Methods

### Study participants

For the analyses of this paper, we used data from 161 individuals enrolled in a previous study in which ERPs were collected on brain reactivity to an array of emotionally arousing images^36^. Participants in the original study lived at a stable address within Harris county, TX, did not report any psychiatric disorder during a screening interview, were between the ages of 18 and 55 years, were able to speak English, had access to a telephone, were negative to a urine drug panel and to a urine pregnancy test. The mean age of the sample was 35 years (SD = 9), 75% of the sample were females and the racial distribution included 37% Blacks, 28% Whites, 14% Asian, 21% others (including not reported).

### Picture viewing task

The images used in the study were selected from the International Affective Picture System (IAPS)^43^ and from a collection used in^44,45^ created by downloading images from the internet and from another libraries of images. The images used for the study belonged to 8 categories (Erotica, Romantic, Food, Neutral, Neutral Objects, Pollution, Attack, Mutilations) with 16 pictures in each condition. To conduct the analyses described in this paper, we excluded images depicting objects (i.e., food, pollution, and neutral objects) and re-classified erotic and mutilation contents as “High arousing” and romantic and attack contents as “Low arousing”. The images were presented for 4 s in pseudo-random sequences (no more than two consecutive pictures of the same condition). A 3–5 s random intertrial interval, showing a white fixation cross against a black background, followed each image. The slideshow lasted approximately 20 min and included four 30 s intervals, during which the participant was instructed to relax. The images were presented on a plasma screen placed approximately 1.5 m from the participant’s eyes. The images subtended approximately a $24^{\circ }$ horizontal viewing angle.

### Data collection procedures

All study procedures for the original study were approved by the UT MDAnderson IRB and were conducted in accordance with the relevant guidelines and regulations. Written informed consent was collected from each participant before starting any procedure. Details about the study are provided in the original publication^36^. Briefly, the study included one in-person laboratory visit when, after obtaining informed consent, eligibility was confirmed and the electroencephalogram (EEG) data were recorded while participants passively looked at a slideshow that included a wide array of images selected from a standardized database. During the slideshow, EEG was continuously recorded using a 129-channel Geodesic Sensor Net, amplified with an AC-coupled high input impedance (200 M$\Omega$) amplifier (Geodesic EEG System 200; Electrical Geodesics Inc., Eugene, OR), and referenced to Cz. The sampling rate was 250 Hz, and data were filtered online by using 0.1 Hz high-pass and 100 Hz low-pass filters. Scalp impedance of each sensor was kept below 50 K$\Omega$, as suggested by the manufacturer. At the conclusion of the visit, participants were debriefed and compensated for their time.

### Data reduction procedures

Eyeblink artifacts were corrected using a spatial filtering method as implemented in the BESA software (BESA GmbH, Grafelfing, Germany), data were re-referenced to the average reference and eyeblinks were corrected. Data were imported into BrainVision Analyzer 2.1 (Brain Products GmbH, Gilching, Germany) and filtered with a high-pass filter of 0.1 Hz (12 dB/octave), a low-pass filter of 30 Hz (12 dB/octave), and a notch filter of 60 Hz. The data were then segmented into 900-ms segments, starting 100 ms before stimulus presentation. The 100-ms interval before stimulus presentation was defined as the baseline and subtracted from every data point in the segments. Artifacts were identified in the segmented data and channels contaminated by artifacts in more than 40% of the segments were interpolated using six neighboring channels. Voltage data averaged from 10 centroparietal sensors (EGI electrodes 7, 31, 37, 54, 55, 79, 80, 87, 106, 129) were used in the analyses, as these channels had shown the highest LPP differences between experimental conditions^36^.

### Empirical bootstrap estimates

For $B = 10,000$ bootstrap iterations, trials were resampled with replacement, stratified by condition, so that each bootstrap sample contained the same number of trials in each condition as the original data. For each bootstrap iteration, mean curves by condition were then computed over the resampled trials. The estimated means by condition and associated confidence intervals were then calculated as the mean and empirical quantiles of the bootstrapped distribution of the means by condition. Empirical bootstrap estimates of the differences in the LPP means by condition were obtained similarly by calculating the average over the LPP window for each of the bootstrapped means by condition. For each pair of conditions, we conclude that the LPP means are significantly different if the 5% empirical quantile of the bootstrap distribution of the difference in the means is positive.

### Random phase-amplitude Gaussian process model

Let $\textbf{y}_1, \dots , \textbf{y}_n$ be *n* realizations of a stochastic process on $[a, b] \rightarrow \mathbb {R}$ observed at *T* evenly spaced points; without loss of generality, we will assume $a = 0, b = 1$ in the sequel. We first consider a general form of a curve registration model, to motivate the construction of the proposed RPAGP model. In curve registration, the aim is to match two or more functions that might exhibit a common shape distorted by function-specific variations referred to as *warpings*^47–50^. The problem of curve registration is typically stated as follows: given two functions, $f_1, f_2$, find the warping function $\gamma$ such that $f_1$ and $f_2 \circ \gamma$ are optimally matched according to some criteria. The general class of warping functions consists of all orientation preserving diffeomorphisms of [0, 1], but in practice the space of warping functions considered is often restricted to some subset of parameterized functions, e.g. linear transformations^51^. To accommodate transformations along both the horizontal and vertical axes, an additional warping function is introduced; in this setting, it is common to consider linear transformations $\gamma _1, \gamma _2$, resulting in the phase-amplitude curve registration problem^52–55^. Accordingly, we assume that the observed data $\textbf{y}_i$ result from a *structural* signal function *f*, composed with trial-specific left- and right-transformations $g_i, h_i$ respectively, plus structured noise $v_i$, as

1 $$\begin{aligned} y_i(t) = (g_i \circ f \circ h_i)(t) + v_i(t), ~~~i=1,\ldots ,n;~~t = k\Delta t,\\ \text {for } k = 0, \dots , T - 1. \end{aligned}$$

Specification of this model requires selecting the form of the transformations $g_i, h_i$, a class of signal functions, and the noise structure. This general form of curve registration model has been explored in the literature for different classes of transformations and signal curves, at various levels of generality^47,51,56^.

In ERP studies, there is often scientific interest in estimating differences in signal amplitude and latency across trials, as these differences have been shown to be associated with cognitive and behavioral outcomes^2,3,17^. This suggests parameterizing the class of transformations as $g_i(x) = \beta _i x$, $h_i(x) = x - \tau _i$, where $\beta _i, \tau _i$ are the trial-specific amplitude and latency respectively. We assume *f* is an element of the reproducing kernel Hilbert space (RKHS) determined by a positive-definite kernel function $\kappa (t', t)$. This allows for the class of signal functions to be tailored to a specific application through the choice of an appropriate kernel and prior distribution over the RKHS. In the present work, we assume Gaussian process priors with radial basis kernel for *f*, which is both sufficiently flexible and computationally tractable for the analysis of ERP data^11^. Lastly, following empirical results in the ERP literature^16,57^, the ongoing background activity is assumed to follow an autoregressive structure. Formally, our proposed RPAGP model can be written as

2 $$\begin{aligned} y_{i}(t)&= \beta _i f (t - \tau _i) + v_i(t), i = 1, \dots , n; t = k\Delta t,\\ \text {for } k = 0, \dots , T - 1 \end{aligned}$$

3 $$\begin{aligned} f&\sim \mathcal{G}\mathcal{P}(0, \kappa )\nonumber \\ v_i(t)&= \sum _{j = 1}^p \phi _i v_i(t - j\Delta t) + {\varepsilon }_i(t)\nonumber \\ {\varepsilon }_i(t)&\overset{iid}{\sim }\mathcal {N}(0, \sigma ^2), \end{aligned}$$

with $\Delta t = 1/(T - 1)$. Similar to the terminology of^53^, we refer to *f* as the *structural signal*.

#### Priors on model parameters

To complete the specification of the RPAGP model, we propose the following priors for the model parameters. Letting $\varvec{\tau }=(\tau _1,\ldots ,\tau _n)$ the vector of latency parameters, the prior for $\varvec{\tau }$ is constructed assuming a normal distribution

4 $$\begin{aligned} \varvec{\tau }&\sim \mathcal {N}\left( 0, \sigma ^2_{\tau }\left( I_{n} - \frac{1}{n+1}\textbf{1}\textbf{1}'\right) \right) , \end{aligned}$$

where the marginal variance $\sigma ^2_{\tau }$ should be chosen according to the plausible range of latency for the given scientific application. If the RPAGP model is implemented with time coded on the unit interval (as in the present work), then it will be necessary to translate the scale of the real latency (in, e.g.  milliseconds) to the corresponding value in [0, 1].

When the trial-specific amplitudes $\beta _i$ can be assumed to be unrestricted on $\mathbb {R}$, it is convenient to adopt the prior $\beta _i \overset{ind}{\sim }\mathcal {N}(\mu _{\beta }, \sigma ^2_{\beta })$, which allows for efficient sampling. Choice of the prior parameters is discussed below. In some applications, it may be necessary to restrict $\beta _i \ge 0$, in which case a truncated normal may be used. As for the GP kernel $\kappa$ and associated priors, these should be selected according to prior beliefs about the structural signal *f*. Throughout this article, we adopt the commonly used squared exponential kernel given by $\kappa _{SE}(t', t) = \exp \left\{ -\frac{\rho ^2}{2}(t' - t)^2\right\}$. The length scale parameter $\rho > 0$ governs the smoothness of the functions in the associated RKHS; we use the prior $\rho \sim Gamma(a_{\rho }, b_{\rho })$, with $a_{\rho }$ and $b_{\rho }$ chosen according to the plausible range of values for the signal length scale. Further details are provided in^58^; an extensive investigation of GP covariance structures in the context of EEG data is given in^11^. As for the *AR* coefficients $\varvec{\phi }=(\phi _1,\ldots ,\phi _p)$, these are given a prior $\mathcal {N}(0, \Sigma _{\phi })$, where $\Sigma _{\phi } = \text {diag}\{\sigma _{\phi _1}^2, \dots , \sigma _{\phi _p}^2\}$, with variance values chosen to concentrate the prior mass in the interval $[-1, 1]$. Finally, the prior for the white noise variance $\sigma ^2$ is $Gamma(a_{\sigma }, b_{\sigma })$, with hyperparameters chosen to place diffuse mass over reasonable values for the error variance for the observed data.

We note that, as formulated thus far, the scales of the $\beta _i$ and *f* are not identifiable due to invariance of the likelihood under transformations of the form $\beta _i \rightarrow r \beta _i, f \rightarrow \frac{1}{r}f$ for any constant $r \ne 0$ applied over all $i = 1, \dots , n$. To resolve this, one may fix a nonzero value for $\beta _i$ for one trial, or alternatively, fix a nonzero value of $f(t^*) = a$ at one point $t^*$. When exploring these options, we found that the former solution may introduce errors or affect the model fit if the selected trial is an outlier or especially noisy relative to the other trials, and therefore generally advocate for the latter solution. In fixing a value of *f*, it is beneficial to choose a time point for which *f* is likely nonzero, and to fix the value close to the value of the expected signal. If the scale of *f* and the observed signals are substantially different, priors for the $\beta _i$’s must be calibrated accordingly. In practice, it is useful to examine the empirical mean to identify a reasonable time point and scale for *f*. In this case, it is suggested to set the prior parameters $\mu _{\beta }$ and $\sigma _{\beta }$ so that there is prior mass over the plausible range of observed scaling factors relative to the mean curve. A sensitivity analysis of RPAGP, conducted on simulated data, indicated robust performances of RPAGP in estimating trial-specific amplitude and latency parameters (see Supplementary Material, Table A.3), suggesting that hyperparameter tuning can be reasonably guided by prior scientific knowledge.

#### Posterior inference via MCMC

Let $\varvec{\theta }= (\varvec{\beta }, \varvec{\tau }, \rho , \varvec{\phi }, \sigma ^2)$ represent the vector of model parameters, with $\varvec{\beta }= (\beta _1, \dots , \beta _n) \in \mathbb {R}^n$ the vector of trial amplitudes, $\varvec{\tau }= (\tau _1, \dots , \tau _n)\in \mathbb {R}^n$ the vector of trial latencies, $\rho > 0$ the length scale parameter of the GP squared exponential kernel for the GP prior, $\phi = (\phi _1, \dots , \phi _p)$ the vector of autoregressive coefficients, and $\sigma ^2$ the error variance. Given *f* and $\varvec{\theta }$, the conditional distribution of *y* can be computed from the multivariate normal conditioning formula as

5 $$\begin{aligned} \varvec{y}_i | f&\sim \mathcal {N}(\varvec{\mu }_{\varvec{y}_i|f}, \Sigma _{\varvec{y}_i|f}), \end{aligned}$$

with $\varvec{\mu }_{\varvec{y}_i|f} = K_{\varvec{y}_if}K_f^{-1}f$ and $\Sigma _{\varvec{y}_i|f} = \Sigma _{\varvec{y}_i} - K_{\varvec{y}_if}^{{'}}K_f^{-1}K_{\varvec{y}_if}$, and where $K_{\varvec{y}_if}(t', t) = \beta _i \exp \{-\frac{\rho ^2}{2}(t' - t - \tau _i)^2\}$ and $\Sigma _{\varvec{y}_i} = \beta _i^2 K_f + \Sigma _{\varvec{v}}$ for $K_f(t', t) = \exp \{-\frac{\rho ^2}{2}(t' - t)^2\}$ and $\Sigma _{\varvec{v}}$ the covariance matrix of an *AR*(*p*) process. The log-likelihood is

$$\begin{aligned} \ell (\theta )&= \sum _{i = 1}^n \left[ \left( \varvec{y}_i - \varvec{\mu }_{\varvec{y}_i|f}\right) '\Sigma _{\varvec{y}_i|f}^{-1}\left( y_i - \varvec{\mu }_{\varvec{y}_i|f}\right) + \log (|\Sigma _{\varvec{y}_i|f}|)\right] + nT\log (2\pi ). \end{aligned}$$

Given that $(f, \varvec{y}) | \varvec{\theta }$ are jointly normal, the closed-form posterior for $f | \varvec{y}, \varvec{\theta }$ is calculated as

$$\begin{aligned} p(f | \varvec{y}, \theta ) \propto p(\varvec{y} | f, \varvec{\theta })p(f | \theta ) = \\ \exp \left\{ -\frac{1}{2}\sum _{i = 1}^n(\varvec{y}_i - \varvec{\mu }_{\varvec{y}_i|f})'{\Sigma _{\varvec{y}_i|f}^{-1}}(\varvec{y}_i - \varvec{\mu }_{\varvec{y}_i|f})\right\}\\ \exp \left\{ -\frac{1}{2}f'K_f^{-1} f\right\} , \end{aligned}$$

that is, $f | \varvec{y}, \theta \sim \mathcal {N}(\varvec{\mu }_{f|\varvec{y}}, \Sigma _{f|\varvec{y}})$, with

$$\begin{aligned} \varvec{\mu }_{f | \varvec{y}} = \Sigma _{f|\varvec{y}}\sum _{i = 1}^n\varvec{y}_i'\Sigma _{\varvec{v}}^{-1}K_{\varvec{y}_if}K_f^{-1} \text{ and } \\ \Sigma _{f | \varvec{y}}^{{-1}} = K_f^{-1}\left( \sum _{i = 1}^n K_{\varvec{y}_if}\Sigma _{\varvec{v}}^{-1}K_{\varvec{y}_if}\right) K_f^{-1} + {K_f^{-1}}. \end{aligned}$$

The procedure in Algorithm 1 produces a chain of *B* draws from the joint posterior of $(\varvec{\theta }, f)$. Given the current parameter values $\varvec{\theta }^{b - 1}$, values for $\varvec{\tau }^{b}, \varvec{\beta }^{b},$ and $\rho ^{b}$ are generated as a series of Metropolis-within-Gibbs steps; alternatively, if $\varvec{\beta }$ is unconstrained, $\varvec{\beta }^{b}$ may be sampled from the closed-form posterior for Bayesian linear regression (BLR). Then, $f^b$ is sampled from the closed-form posterior conditional on $\varvec{\tau }^{b}, \varvec{\beta }^{b}, \rho ^{b}$. The residual activity is calculated by removing the trial-specific mean $\varvec{v}^b_i = \varvec{y}_i - \mathbb {E}[\varvec{y}_i | \varvec{\theta }^{b}]$, from which draws of $\varvec{\phi }^{b}, \sigma ^{2, b}$ are made using closed-form posterior updates. We note that in general this procedure requires $\mathcal {O}(T^3)$ operations for inverting $K_f$ and $\Sigma _{\varvec{v}}$, but scales linearly with *n*, and is thus usually computationally feasible for real data from ERP experiments.

**Algorithm 1:**
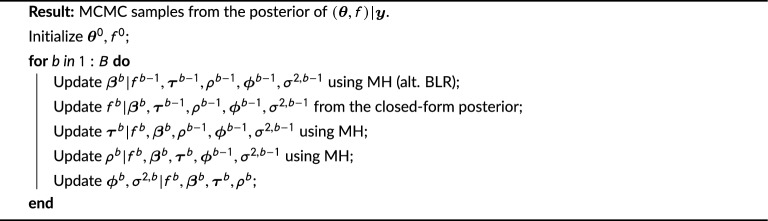
Sampling algorithm.

Given the output of Algorithm 1, inference may be conducted through analysis of the posterior samples $\{\varvec{\theta }^b\}_{b = 1}^B$. In particular, testing for differences in mean latency and amplitude across groups of trials (i.e., experimental conditions) can be carried out directly from the posterior samples of $\varvec{\beta }$ and $\varvec{\tau }$, as we show in the simulations below. Furthermore, the posterior draws of the structural signal *f* can be used to provide visualization of the estimated signal shape and posterior variability, as described below.

#### Inference on trial-level ERP signals

An important feature of the output from our model is the availability of samples on the structural signal *f*, which in turn allows us to produce posterior samples of trial-specific signals. Let $\varvec{\tau }^b, \varvec{\beta }^b$ and $f^b$ be the *b*th MCMC draws of the respective parameters. Model predictions $\hat{y}_i(t)$ of the individual trials can then be obtained as, e.g., the pointwise-median of the posterior samples of the signal for trial *i* calculated as $\hat{y}^b_i(t) = \beta _i^b f^b(t - \tau _i^b)$. Here, however, we focus on the *temporally-aligned* trial estimates $\tilde{y}_i(t) = \widehat{\beta _i f}(t)$, calculated as the median of the posterior samples $\tilde{y}_i^b(t) = \beta _i^b f^b(t)$. This effectively treats the estimated latencies $\hat{\tau }_i$ as nuisance parameters, allowing analysis of features of interest adjusting for trial-specific latency. In our application, preference for the aligned estimates follows from the scientific assumption that the LPP window (see Fig. 1) should be adjusted for each trial according to the trial-specific latency. The aligned estimates may also be averaged to compute $\tilde{f}=\widehat{\bar{ \beta } f}$, the structural signal scaled by the mean amplitudes of the trials (possibly within each condition), which is the most “natural” scaling of *f* relative to the data.

#### Inference on LPP means

Next, we present inference on differences in LPP means across categories using the RPAGP fit. Let us denote the LPP mean as $\mu _{LPP}$. When referring to the parameter for a specific stimulus condition, e.g. Highly arousing, we will denote this as $\mu _{LPP}^{High}$. To estimate the LPP means, we produce the aligned trial estimates $\tilde{\varvec{y}}_i^b$ from the *b*th MCMC draw and compute the corresponding LPP means by averaging the $\tilde{\varvec{y}}_i^b$ within each condition, and then averaging over the LPP window. That is, for condition $c \in \{High, Low, Neutral\}$, trial conditions $c_i$, $n_c$ the number of trials in condition *c*, and LPP window $[t_{lwr}=300, t_{upr}=700]$ with length $T_{LPP} = t_{upr} - t_{lwr}$, the *b*th posterior draw of the LPP mean is

$$\hat{\mu }^{c, b}_{LPP} = \frac{1}{n_c T_{LPP}} {\sum _{t = t_{lwr}}^{t_{upr}}} \sum _{i:c_i = c} \tilde{y}_i^b(k\Delta t)$$

and the posterior distribution of the differences of means can then be calculated as $\hat{\mu }^{c_1, b}_{LPP} - \hat{\mu }^{c_2, b}_{LPP}$, for conditions $c_1, c_2$. Similarly, an *LPP* mean for a single trial *i*, is calculated as $\hat{\mu }^{i, b}_{LPP} = \frac{1}{T_{LPP}} {\sum _{t = t_{lwr}}^{t_{upr}}} \tilde{y}_i^b(k\Delta t)$.

Testing of the LPP hypotheses for differences in conditions is conducted by computing the 5% lower credible bound for the mean differences, and concluding significance if this value is positive. In comparing performance of RPAGP and frequentist methods, we note that while the bootstrap confidence intervals and Bayesian credible intervals have different theoretical foundations, it has been shown that Bayesian CIs approximately satisfy frequentist coverage properties under fairly general conditions. Further discussion is provided in^59^.

#### Parameter settings

Considering the choice of priors and hyperpriors, our model has a total of $6 + p$ free parameters, for autoregressive order *p*, which reduces to 7 parameters if all autoregressive coefficient priors are chosen to be the same. The parameters and hyperparameters that must be set to initialize the model are: (i) the variance of the latency paramaters; (ii) the variance of the amplitudes; (iii) the hyperprior scale and shape for the length scale of the structural signal; (iv) the prior for the autoregressive noise parameters, including the choice of the AR order; and (v) the hyperprior scale and shape for the variance of the white noise. In all analyses of this paper, prior hyperparameters were chosen to specify weakly informative priors consistent with the assumptions and available prior information from existing ERP studies. In order to ensure identifiability of the $\varvec{\beta }$ parameters, we chose to fix the value of the structural signal 400 ms after stimulus presentation to be approximately equal to the value of the grand average at this time point. We consequently chose the prior $\beta _i \overset{ind}{\sim }\mathcal {N}(1, 0.5)$ to place the majority of the prior mass on (0, 2), $\sigma _{\tau }^2 = 0.02$ to put approximately 95% of marginal prior mass for latencies equal to $\pm 9$ time points, or shifts of $\pm 4$% of the LPP window, and $a_{\rho } = 12, b_{\rho } = 1$ to give a wide range of plausible GP length scales. The autoregressive order for *v* was selected as $p = 2$ after examination of the autocorrelation and partial autocorrelation functions of data residuals resulting from subtraction of the subject-specific average ERPs and the residuals from RPAGP fit with white noise error; prior variance for $\phi _1, \phi _2$ was set at 0.5. The prior for $\sigma ^2$ was set as *Gamma*(1, 1).

The RPAGP model was fit via Algorithm 1 for $B = 8000$ MCMC draws. On a MacBook Pro computer with 2 GHz Quad-Core Intel Core i5 and 16 GB RAM, this took about 10 hours, for one subject. Analyses on multiple subjects were run in parallel on a cluster computer. For Metropolis-Hastings sampling, Gaussian proposal distributions centered at the previous parameter draw were used, with variances set at 0.005 and 1 for $\varvec{\tau }$ and $\rho$, respectively. Convergence of all parameters was assessed by the Gelman-Rubin diagnostic measure $\hat{R}$, with sampling terminated when all parameters yield $\hat{R} < 1.1$ as recommended in^60^. Effective sample sizes for all parameter posterior samples were also calculated following termination and evaluated according to the stopping rule in^61^ as an additional check for any issues with sampling.

### Data quality measures

To quantify the practical effect of the model de-noising provided by RPAGP in the LPP experiment, we consider the signal-to-noise ratio (SNR), here defined as the ratio of the point estimate LPP mean for condition *c* to the 90% credible interval width for the estimate, that is $SNR = \frac{\hat{\mu }^c_{LPP}}{CI_{upr} - CI_{lwr}}$^62^. The SNR quantifies the magnitude of the signal relative to the variability of the noise in the data, i.e., a larger SNR indicates a more easily detected signal. When computing SNR for difference in LPP means between different experimental condition, we evaluated the SNR as the ratio of the absolute value of the difference LPP means for condition $c_1$ and $c_2$ to the corresponding 90% credible interval width, namely SNR = $\frac{\hat{\mu }_{LPP}^{c1,c2}}{CI^{c1,c2}_{upr} - CI^{c1,c2}_{lwr}}$, with $\hat{\mu }_{LPP}^{c1,c2} = |\hat{\mu }^{c_1}_{LPP} - \hat{\mu }^{c_2}_{LPP}|$

An alternative measure of (subject-level) data quality is the standardized measurement error (SME) proposed by^46^. The SME of a sample *y* relative to a statistic of interest *R*(*y*) is defined as the sampling standard deviation, which can be estimated via a bootstrap resampling procedure. In our context, with the LPP mean $\hat{\mu }_{LPP}$ as the statistic of interest, the SME is $SD(\hat{\mu }^{c_1}_{LPP} - \hat{\mu }^{c_2}_{LPP})$, where $c_1, c_2$ refer to two experimental conditions, e.g., High and Low arousing images. A lower SME score indicates lower variability of the test statistic across trials.

### Power analysis

We assessed the power of the RPAGP method for testing the three hypotheses implied by $\mu ^{High}_{LPP}>\mu ^{Low}_{LPP} > \mu ^{Neutral}_{LPP}$, for varying number of trials, as follows. In order to mimic the relative sizes of the categories in the original sample, for total number of trials $n = 15; 25$, we resampled with replacement 0.4*n* trials from each of the High and Low conditions, and 0.2*n* trials from the Neutral condition, to create 20 synthetic data sets for each sample size. We then conducted the test for difference of LPP means, concluding significance if the 5% quantile of the posterior distribution of the difference of the means is positive. The estimated power is computed as the proportion of significant tests. Similarly, for comparison, we conducted the empirical bootstrap test for differences in means by conditions. For each synthetic data set, we resample with replacement to create $B = 10{,}000$ bootstrap data sets. On each of these data sets, we compute the *LPP* means by condition and pairwise differences in means, concluding a significant difference when the 5% quantile of the bootstrap distribution of difference of means is positive.

### Supplementary Information

Supplementary Information.

## Data Availability

ERP data supporting the findings of this study are available from the corresponding author upon reasonable request.

## Code availability

Code implementing the Bayesian RPAGP model described in the “Methods” section of this paper can be downloaded from GitHub at https://github.com/Beniamino92/BayesRPAGP/.
