# Supplementary material for: Improved data quality and statistical power of trial-level event-related potentials with Bayesian random-shift Gaussian processes
Source: Sci Rep. 2024 Apr 17;14:8856. doi: 10.1038/s41598-024-59579-2 (PMC11024164; doi:10.1038/s41598-024-59579-2)
Supplement: Supplementary file 1 — Supplementary Information. [file 41598_2024_59579_MOESM1_ESM.pdf]

ORIGINAL ARTICLE

Journal Section

Improved Data Quality and Statistical Power of Trial-Level Event-Related Potentials with Bayesian Random-Shift Gaussian Processes

Dustin Pluta[†, 1] | **Beniamino Hadj-Amar**[†, 2] | **Meng Li**[2] | **Yongxiang Zhao**[3] | **Francesco Versace**[4] | **Marina Vannucci**[*,2]

A | SUPPLEMENTARY MATERIAL

A.1 | Performance assessment on simulated data

We use simulated data to further investigate performance of the proposed RPAGP model to reconstruct the structural signal. First we simulated data that follow the general structure suggested by the real ERP data of our application. In particular, we generated data from our model (Eq. (2) in the main paper) for two categories labeled A and B. The common structural signal f was drawn from $\mathcal{GP}(0, \kappa_{SE})$ with $\rho = 10$, realized at $T = 50$ points. We generated half of the trials with amplitude parameters drawn as $\beta_{i:c_i=A} \sim \mathcal{N}(1, \sigma_\beta)$ and half as $\beta_{i:c_i=B} \sim \mathcal{N}(1.5, \sigma_\beta)$. Latency parameters were drawn from the same distribution for all trials as $\tau_i \sim \mathcal{N}(0, \sigma_\tau)$. Ongoing activity $\boldsymbol{v}_i$ was generated as an $AR(2)$ process with coefficients $\phi_1 = 0.5, \phi_2 = 0.1$ and white-noise variance σ_ε. We investigated performances for different values of the parameters $\sigma_\beta, \sigma_\tau$ and σ_ε. Figure A.1 shows the true structural signal f and the simulated trial-level data for one dataset simulated with $\sigma_\beta = 0.1, \sigma_\tau = 0.01$ and $\sigma_\varepsilon = 0.1$ and $n = 30$.

In all cases, we set hyperparameters to specify weakly informative priors, similar to those chosen in the real ERP data application. In order to ensure identifiability of the $\boldsymbol{\beta}$ parameters, we fixed $f(0.5) = m$, with m chosen as the empircal mean of all trials at this time. We consequently chose the prior $\beta_i \stackrel{ind}{\sim} \mathcal{N}(1, 0.1)$ to place the majority of the prior mass on $(0, 2)$, $\sigma_\tau^2 = 0.01$ to put approximately 95% of marginal prior mass for latencies equal to ± 10 time points, or shifts of $\pm 20\%$ of the time window in Figure A.1, and $a_\rho = 12, b_\rho = 1$ to give a wide range of "plausible" GP length scales. The RPAGP model was fit via the algorithm described in the main paper, with $B = 3,000$ MCMC draws. On a MacBook Pro computer with 2 GHz Quad-Core Intel Core i5 and 16 GB RAM, this took about 30 minutes, for each replicated dataset. Proposal distribution variances were set at 0.001 and 1 for $\boldsymbol{\tau}$ and ρ, respectively. Convergence of all parameters was assessed by inspecting the Gelman-Rubin diagnostic measure $\hat{R}$ and the effective sample sizes for all parameter posterior samples.

The RPAGP estimate $\tilde{f} = \widehat{\bar{\beta} f}$, computed as the posterior median of the distribution of the temporally-aligned trial estimates, for one dataset simulated with $\sigma_\beta = 0.1, \sigma_\tau = 0.01$ and $\sigma_\varepsilon = 0.1$ and $n = 30$ is shown in Figure A.1. It is evident that, for this example data, the empirical mean tends to be attenuated toward 0 relative to the structural signal, due to the presence of random latencies across the trials. By adjusting for the trial-specific latencies, the model is able to provide a more accurate estimate of the true structural signals relative to the empirical means.

Next, we investigated performances on replicated datasets. For each simulated dataset, we estimated the scaled

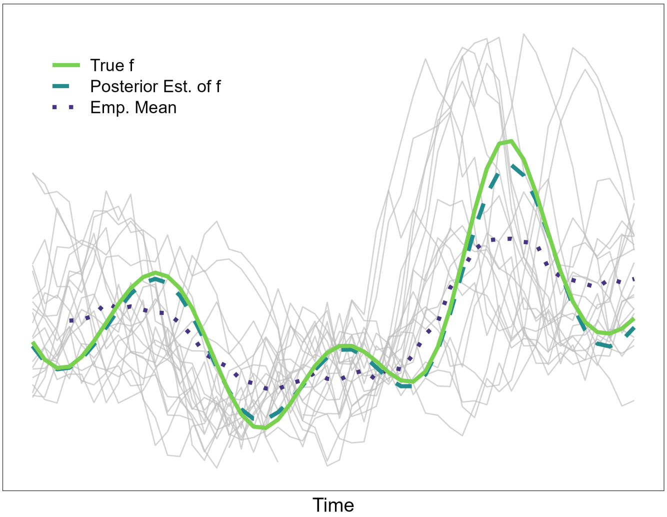

FIGURE A.1 **Simulation study:** Example of data generated from our model (Eq. (2) in the main paper). The true structural signal f (solid) is accurately recovered by the RPAGP estimate $\tilde{f} = \widehat{\beta f}$ (dashed). By comparison, the peaks of the empirical mean (dotted) show substantial flattening relative to the true f due to the latency present in the trial data.

structural signal $\tilde{f}$ and calculated the L_2 error for the model estimate as $Err_f^2(\tilde{f}) = \frac{1}{T}\sum_{t=1}^{T}(f(t) - \tilde{f}(t))^2$. We considered 35 replicates for each simulated scenario and computed mean squared errors (MSEs) by averaging the squared L_2 error across the 35 replicates. Results are reported in Table A.1 where we also show the MSEs obtained by estimating the true structural signal by the empirical mean of the raw data. Results clearly indicate that the RPAGP reconstruction of the true signal is stable with respect to the latency variance, whereas the accuracy of the empirical mean deteriorates drastically as the variance increases. The empirical mean and model estimate are similarly accurate when there is no trial-specific latency ($\sigma_\tau = 0$); when some latency is present ($\sigma_\tau = 0.1$), the model consistently outperforms the empirical estimate; when the variability of the latency across trials is increased ($\sigma_\tau = 0.1$), the model estimate remains relatively accurate while the empirical estimate continues to degrade.

A.2 | Two-group power analysis on simulated data

For further investigation, we performed a two-group power analysis on simulated data with varying numbers of trials and by generating data with different signal-to-noise ratios. The simulation design and settings follow those used for Table A.1, with data generated with half of the trials having amplitude from $\mathcal{N}(1, \sigma_\beta)$ and half from $\mathcal{N}(1.5, \sigma_\beta)$, representing data from two different experimental conditions. For each replicate of this simulation, we drew a component curve $f \sim \mathcal{GP}(0, \kappa_{SE})$, realized at $T = 50$ points. From f, we created n signals by sampling trial-specific latencies $\boldsymbol{\tau} \sim \mathcal{N}(\boldsymbol{\mu}_\tau, \sigma_\tau^2 I_n)$ and amplitudes $\boldsymbol{\beta} \sim \mathcal{N}(\boldsymbol{\mu}_\beta, \sigma_\beta^2 I_n)$, where $\boldsymbol{\mu}_\tau$ and $\boldsymbol{\mu}_\beta$ are vectors of length n. The synthetic data y_i for trial i was then generated from our model (Eq. (2) in the main paper) by adding $AR(2)$ noise $\boldsymbol{v}_i$ and white noise ε_i to the signal $\beta_i f(t - \tau_i), i = 1, \ldots, n$. Each setting was replicated 35 times. For convenience in displaying results, we calculate a signal-to-noise ratio for each simulation setting as $SNR = \frac{\Delta\beta}{\sigma_\beta + 2\sigma_\tau}$, where $\Delta\beta$ is the difference in mean amplitudes between the two groups. This gives an approximate measure of the difficulty of the test in each setting.

σ_β	σ_τ	σ_ε	Emp. MSE	RPAGP MSE
0.01	0	0.1	0.051 (0.025, 0.187)	0.050 (0.023, 0.265)
0.1	0	0.1	0.064 (0.017, 0.163)	0.050 (0.015, 0.179)
0.2	0	0.1	0.057 (0.013, 0.121)	0.047 (0.012, 0.091)
0.01	0.1	0.1	0.108 (0.054, 0.208)	0.061 (0.002, 0.294)
0.1	0.1	0.1	0.086 (0.042, 0.222)	0.065 (0.008, 0.302)
0.2	0.1	0.1	0.095 (0.054, 0.231)	0.106 (0.021, 1.387)
0.01	0.2	0.1	0.257 (0.056, 0.819)	0.115 (0.009, 0.571)
0.1	0.2	0.1	0.239 (0.103, 0.581)	0.113 (0.003, 0.519)

TABLE A.1 **Simulation study:** Results for data generated with half of the trials having amplitude from $\mathcal{N}(1, \sigma_\beta)$ and half from $\mathcal{N}(1.5, \sigma_\beta)$, latency parameters drawn for all trials as $\tau_i \sim \mathcal{N}(0, \sigma_\tau)$ and AR(2) ongoing activity with white-noise variance σ_ε.

			EMP			RPAGP		
σ_β	σ_τ	1/SNR	$n = 10$	$n = 20$	$n = 30$	$n = 10$	$n = 20$	$n = 30$
0.01	0	0.02	0.35	0.35	0.35	0.77	0.89	0.94
0.1	0	0.2	0.35	0.35	0.35	0.86	0.91	0.91
0.2	0	0.4	0.35	0.35	0.35	0.97	0.89	0.94
0.01	0.1	0.42	0.30	0.25	0.30	0.69	0.83	0.83
0.1	0.1	0.6	0.30	0.20	0.25	0.71	0.89	0.83
0.2	0.1	0.8	0.30	0.20	0.25	0.80	0.83	0.80
0.01	0.2	0.82	0.30	0.20	0.20	0.34	0.74	0.74
0.1	0.2	1.0	0.30	0.20	0.15	0.49	0.69	0.77

TABLE A.2 **Simulation study:** Estimated power for RPAGP and empirical tests for group differences. The signal-to-noise ratio (SNR) approximately quantifies the difficulty of the test setting at a given sample size. Power was calculated from 35 replicates per simulation setting. In most cases, RPAGP shows substantially greater power than the empirical method.

a_ρ	σ_τ	μ_β	β Ratio Err.	$MSE_{\boldsymbol{\tau}}(\hat{\boldsymbol{\tau}})$
10	0.1	0.5	0.307	0.015
10	0.1	1	-0.155	0.016
10	0.1	2	-0.0937	0.016
10	0.1	10	0.663	0.017
10	0.05	1	0.168	0.0127
10	0.25	1	0.159	0.0135
10	0.5	1	0.199	0.0142
10	1	1	0.203	0.0146
1	0.1	1	0.201	0.0144
5	0.1	1	0.465	0.0151
20	0.1	1	1.02	0.0152

TABLE A.3 **Simulation study:** Errors for estimating $\boldsymbol{\beta}$ and $\boldsymbol{\tau}$ when varying the prior mean of the amplitudes μ_β, the prior variance of the latency σ_τ, and the prior shape parameter a_ρ of the GP length scale ρ.

The model based test of difference across conditions was conducted by computing the 95% posterior credible interval of the difference in mean amplitude between the two groups, and concluding significance if the interval does not include zero. For comparison, we conducted an empirical test by computing a 95% bootstrap confidence interval of the difference in global means between the two groups; for each bootstrap replicate, this was obtained by first computing the empirical group mean signals, taking the average over time of the group means, and taking the difference of these group mean averages. This test procedure is similar to methods used in the ERP literature for detecting amplitude-based group differences [1, 2]. The estimated powers and simulation settings are given in Table A.2. The results show that the power of the RPAGP test is substantially greater than that of the empirical method in the majority of simulation settings considered, and is universally better than the empirical method for the largest sample size $n = 30$.

A.3 | Sensitivity analysis

Finally, we used simulated data to evaluate the sensitivity of the model with respect to the specification of the priors, by repeating the previous simulation for varying choices of prior hyperparameters for $\rho, \boldsymbol{\tau}$, and $\boldsymbol{\beta}$. Specifically, we considered the following changes: the prior $\rho \sim Gamma(a_\rho, 1)$ for various choices of a_ρ; the prior $\beta_i \sim \mathcal{N}(\mu_\beta, \sigma_\beta)$ varying μ_β; the prior for $\boldsymbol{\tau}$ for varying σ_τ. Results are shown in Table A.3. Due to the unidentifiable scale of the β parameters, estimation accuracy for $\boldsymbol{\beta}$ is computed in terms of the error the ratio of mean amplitudes between the two groups to the true ratio of group amplitudes, i.e. $\bar{\beta_B}/\bar{\beta_A} - \mu_B/\mu_A$, which is then averaged over simulation replicates. Estimation accuracy of the parameters τ is reported as the mean squared error in estimating the trial-specific latencies $MSE_{\boldsymbol{\tau}}(\hat{\boldsymbol{\tau}}) = \frac{1}{n}\sum_{i=1}^{n}(\hat{\tau}_i - \tau_i)^2$, averaged across simulation replicates for each setting.

The RPAGP estimation is stable with respect to changes in the prior shape parameter, with some increase in the error of the estimated parameters observed for $\mu_\beta = 10$. For these settings, the model is robust to changes in σ_τ, with essentially no difference in errors across the parameter values considered. The model is mostly insensitive to changes

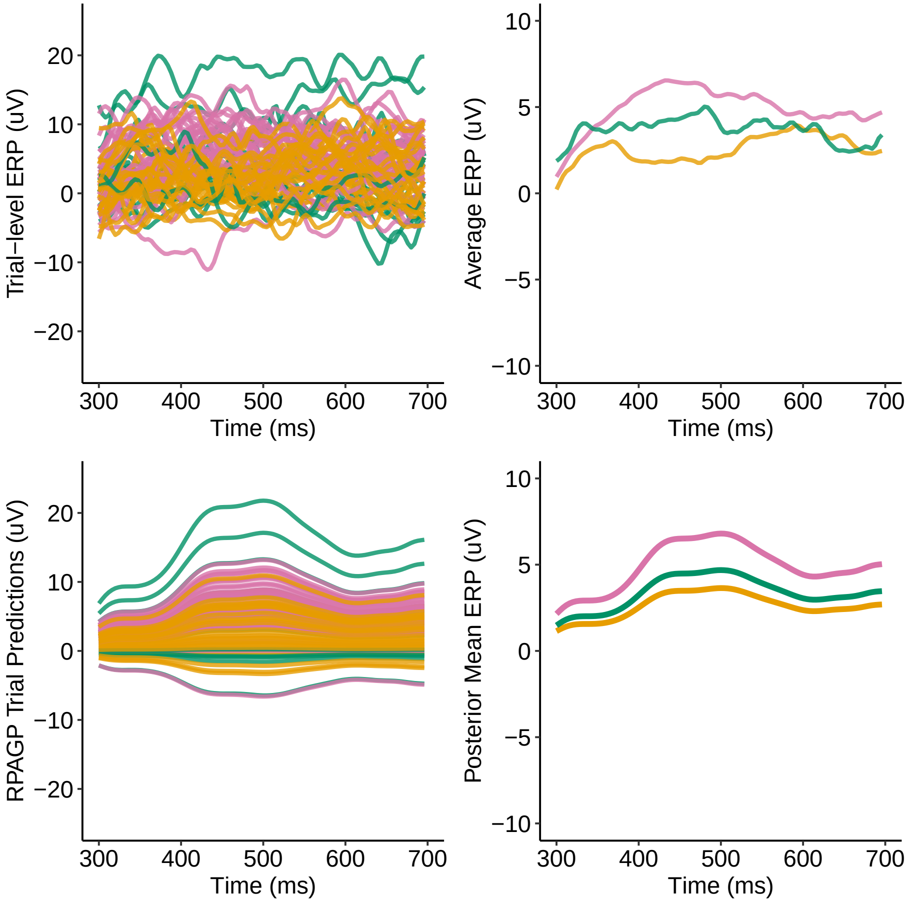

FIGURE A.2 (top) Raw trial data from a single subject and means by condition. (bottom) Temporally-aligned RPAGP trial predictions, estimated via the posterior median, and means by condition obtained by averaging the trial estimates.

in the length scale shape parameter, but shows an increase in the estimation of the amplitude ratio for $a_\rho = 20$.

A.4 | Additional results on real ERP data

Figures A.2, A.3, A.4 and A.5 show raw trial data and means by condition (top), together with the temporally-aligned RPAGP trial predictions and the means by condition obtained by averaging the trial estimates (bottom), for 4 subjects, two of them satisfying the expected LPP mean relationships among conditions and two who do not.

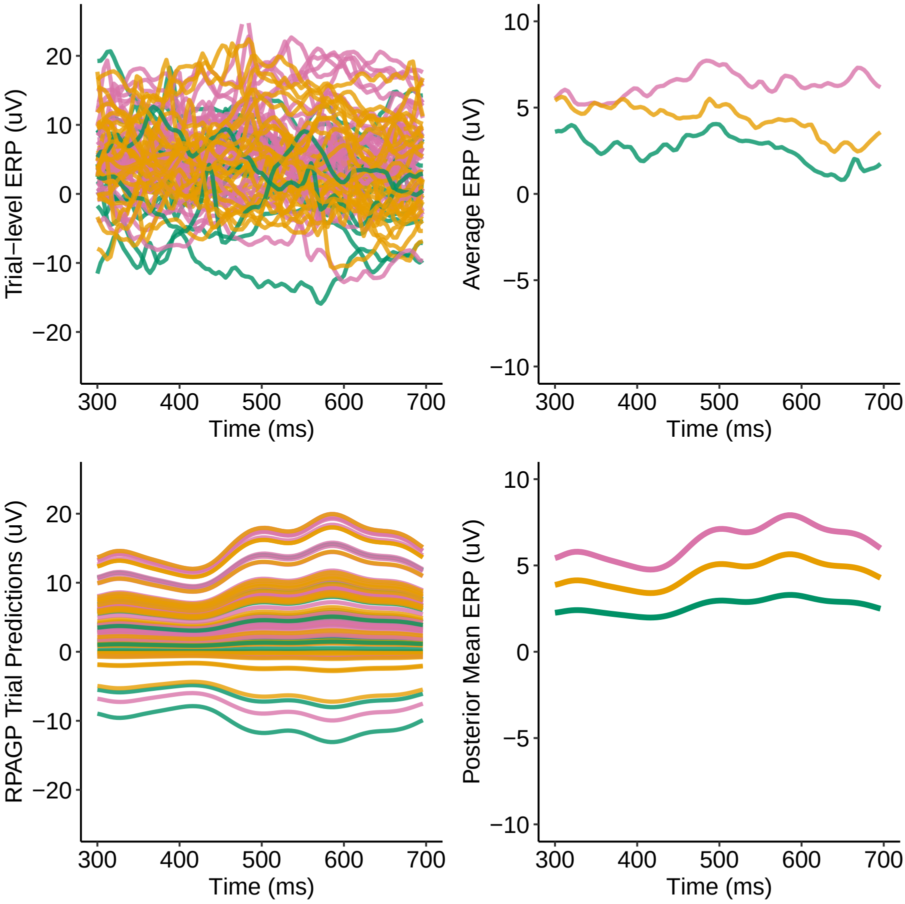

FIGURE A.3 (top) Raw trial data from a single subject and means by condition. (bottom) Temporally-aligned RPAGP trial predictions, estimated via the posterior median, and means by condition obtained by averaging the trial estimates.

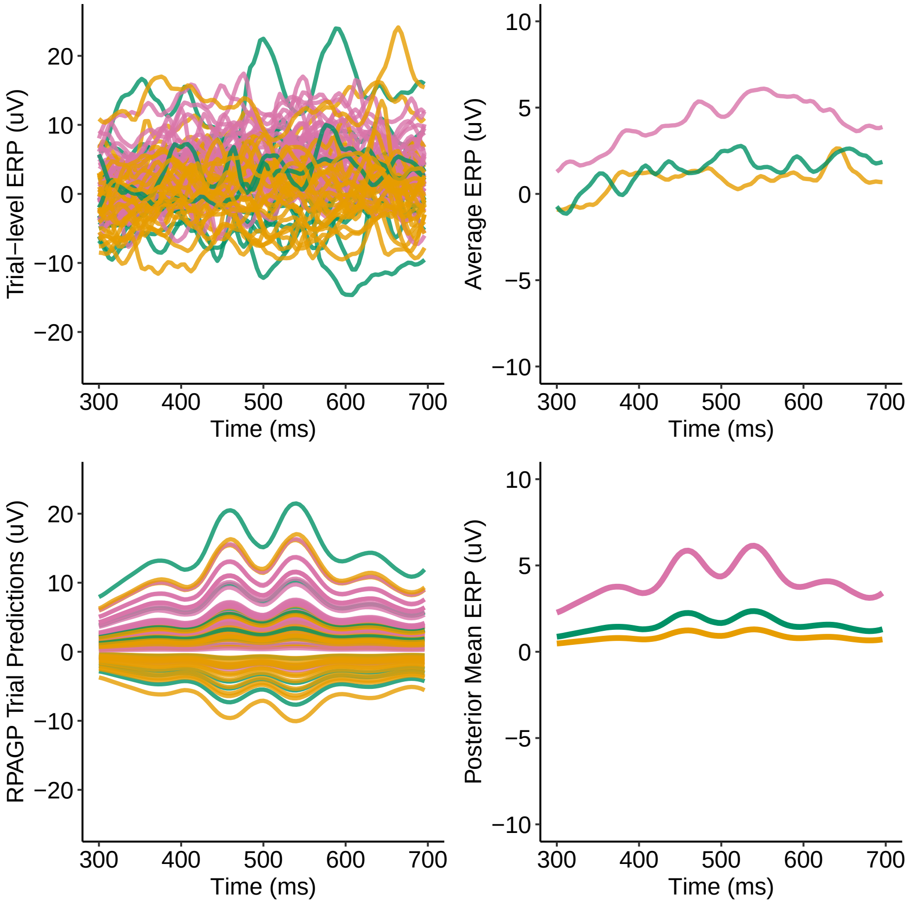

FIGURE A.4 (top) Raw trial data from a single subject and means by condition. (bottom) Temporally-aligned RPAGP trial predictions, estimated via the posterior median, and means by condition obtained by averaging the trial estimates.

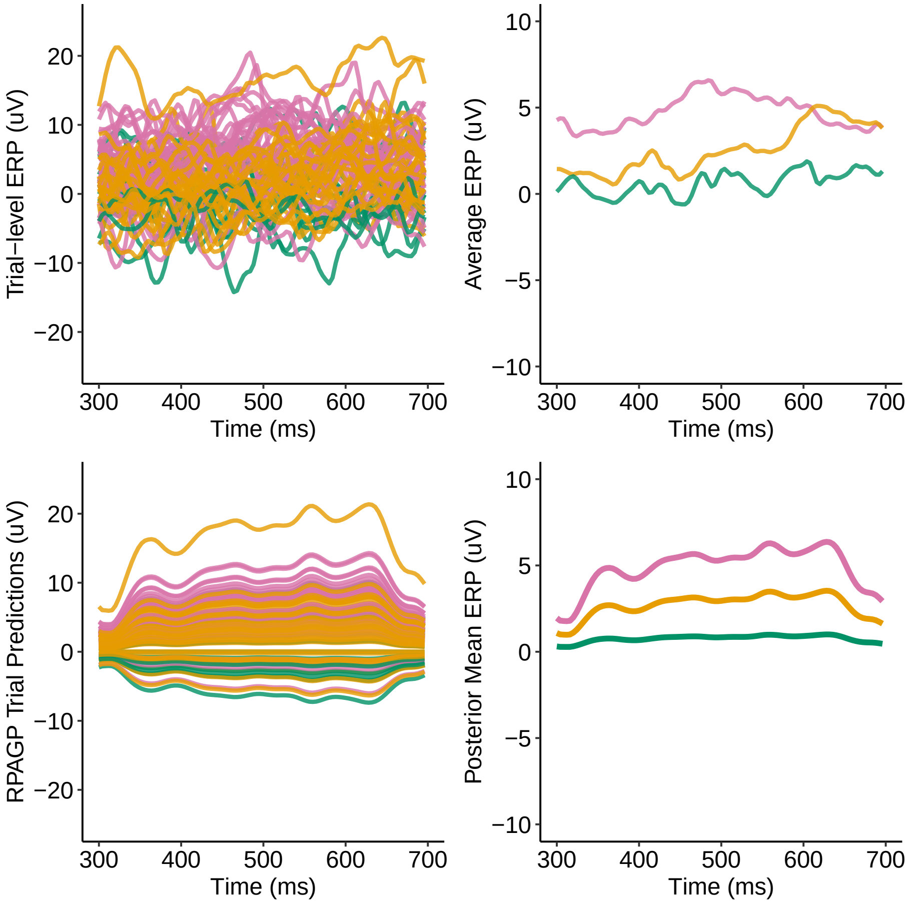

FIGURE A.5 (top) Raw trial data from a single subject and means by condition. (bottom) Temporally-aligned RPAGP trial predictions, estimated via the posterior median, and means by condition obtained by averaging the trial estimates.

References

[1] Rousselet GA, Husk JS, Pernet CR, Gaspar CM, Bennett PJ, Sekuler AB. Age-related delay in information accrual for faces: evidence from a parametric, single-trial EEG approach. Bmc Neuroscience 2009;10(1):1–20.

[2] Abootalebi V, Moradi MH, Khalilzadeh MA. A comparison of methods for ERP assessment in a P300-based GKT. International Journal of Psychophysiology 2006;62(2):309–320.
